# Seasoned Veterans of the Waiting Room: Stories from Working-Class Neighbourhoods of Delhi, India

**DOI:** 10.1007/s11013-025-09934-3

**Published:** 2025-11-27

**Authors:** Kavita Dasgupta

**Affiliations:** Max Planck Institute for Social Anthropology, Advokatenweg 36, 06114 Halle (Saale), Germany

**Keywords:** Care, Communities, Storytelling, Uncertainty, India

## Abstract

Taking seriously the idea that liberation medicine should include, or even start from, the perspectives of those who are at the margins of the current medical system, this article draws on the perspective provided by a group of working-class writers who inhabit these margins, through an ethnographic story about medical failure and radical care. The story follows a middle-aged couple, Farid and Shakeela, residents of a working-class neighbourhood in Delhi, who travel to one of the largest public hospitals in the city for the delivery of their baby. Upon arrival, they encounter a hospital as a separate world, a space with its own rules and language, with maze-like pathways. Through the experiences of this couple, this article sheds light on some questions that help to theorize liberation medicine: In a medical system which is vast and overwhelming, does a labourer have any agency in finding a toe hold, or are they completely helpless and dependent on doctors, nurses, and technicians? Doctors diagnose an illness; however, who defines sickness? What makes a daily wage earner determine the condition of their health? Through reading this story, as well as the pedagogy of its writers, this article sheds light on the significance of networks of care which are easy to overlook whilst also calling for perfect institutions.

## Through the Hospital Gates

*Farid* 1 *and Shakeela walked up to the SKP* 2 *Hospital gates. Shakeela was heavily pregnant with her third child. Her first two had been born at home. Two beautiful girls. But this third one had already begun giving her trouble. So, in her heart she had begun referring to him as a boy! It was the month of October and the sun had mellowed in the sky. Farid had a spring in his step this morning, and Shakeela waddled behind him, rushing to keep pace. She was filled with anticipation and a twinge of nervousness. Shakeela’s girls had been delivered at home by the midwife of the local government dispensary. She had known her for many years now. The doctor at the dispensary was also known to them. He was strict and would scold her often. But he was also kind. He would always reprimand Shakeela for working too much and not taking her vitamins when she complained about feeling weak. As time approached for her delivery, the doctor had advised her to go to the hospital for the birth of this baby. Shakeela was older now, had been extremely weak through this pregnancy, and was also anaemic. So, Farid had taken leave from his tailoring shop, placed his white skull cap on his head, and announced that it was time to take Shakeela to the hospital. Shakeela and her daughters had quickly sprung into action. They had packed a little tiffin box* 3 *with some roti* 4 *and vegetables, filled an old cola bottle with water, and her older daughter had also slipped in a couple of her Ammi’s favourite orange candies. Farid had taken out the local doctor’s referral, which had been neatly spread out under the mattress of his bed, folded it into two and put it in the front pocket of his shirt, and walked out of the house, calling out to his wife. Shakeela had held on to her daughters’ small hands and hugged their little bodies. “Ammi, come back soon with our baby brother,” they said as they waved bye-bye to her.*

*As Farid Bhai and Shakeela approached the vast hospital complex, they were inundated with noise and the fast-paced movement all around. SKP Hospital is one of the largest public hospitals of North India. People come there from all corners of Delhi, from neighbouring towns as well as from distant cities and villages. The road outside the hospital is full of rushing traffic, large buses, trucks, cars, scooters, tuk-tuks,* 5 *and cycles. The entrance is flanked on all sides with food vendors selling fresh fruits, chopped papaya, coconut slivers, and roasted sweet potatoes. Stalls fry out snacks for patients and their families. Young boys weave in and out of the crowd selling bottles of water and cloth masks. Milling all around are thousands of people walking in and out of the large iron hospital gates. The constant din of traffic and people talking is interspersed with sounds of the ambulance siren.*

*Shakeela moved closer to her husband, and he extended his hand and caught hers in a tight grasp. The front lawns of the SKP Hospital swelled with a crowd that filled up every corner. The area between the “Emergency” and the “Out Patient Department” (OPD) seemed to be the best for those who have a long wait, as it provided some space for people to rest. Farid found a spot for Shakeela to sit and set off to figure out what to do and where to go next. “Hold on tightly to your phone and purse,” he told her as he hurried off into the crowd.*

*Shakeela looked around, overwhelmed. There were people everywhere. Some walking past her purposefully whilst others, just like her, were sitting on the pavement, on benches, in the garden or under the trees. Announcements were being made constantly over loudspeakers, asking patients and families to take care of their possessions, be vigilant of pickpockets, and providing a constant stream of instructions: which window or counter to approach in which case. It was too much information for Shakeela to absorb in the din and chaos of her surroundings. Perhaps Farid would fare better, she thought. As she looked around, she saw a hospital staff member mingling amidst the waiting patients and their families. This heavyset man was moving around with such gentleness in the entire open area. He would stop and talk with people, ask them how they were, and then look at all their documents. Perhaps he came often, as many patients seemed to know him. He was wearing a mask, a cap over his head, and gloves on his hands. Yet most people recognized him. Some people would approach him just because he was dressed in hospital scrubs. A man sitting close to Shakeela called out, “Compounder* 6 *sahib,* 7 *please come here.” Compounder sahib came up to the man, looked at his papers, and told him to go to the tuberculosis ward, but first he should go and get registered. He pointed out the relevant buildings and told him where to go. He then looked at Shakeela and asked for her documents. Shakeela was totally taken aback at being addressed and just shook her head. Compounder sahib silently pointed out the “Maternity Block” and walked on to the next person. Shakeela stood up. She had stuffed her phone inside her jumper. She quickly took it out and called Farid with a great sense of achievement.*

This story, about Farid Bhai^8^ and Shakeela, was written by the Ankur Writers Collective from Delhi.^9^ We have just read its beginning and will follow their story throughout this article. The Ankur Writers Collective is an initiative of Ankur, an NGO that offers “alternatives in education” to young people from working-class communities of Delhi. For several years now, Ankur has formed a partnership with scholars from the Max Planck Institute for Social Anthropology and Leipzig University in Germany. This collaboration has led to a series of texts about health care, housing, and e-governance, some of which are currently being published on the multimodal website “Ticketless Travellers” (https://ticketless-travellers.info). Farid and Shakeela’s story is one of the many compendiums produced during this partnership.

The process of how these stories were collaboratively authored gives us an insight into how they should be read as ethnographic texts. Each story begins with the author conducting research in their localities. They have been trained by mentors at Ankur to draw upon their everyday experiences, specifically of their own neighbourhoods, which are largely inhabited by labourers, vegetable vendors, cleaners, rickshaw pullers, and domestic workers, people who must earn daily wages to meet the basic needs of their life. Once the research is completed, the authors start developing the stories, which then go through an intensive cycle of feedback and review before they take their final form. The first reviewers are the subjects of the research themselves, who ensure that their experiences have been narrated without any factual discrepancies or errors of representation. After this, the stories go through rounds of peer review amongst the young writers themselves, when they are read repeatedly and every detail is perused and debated. Sometimes, two or more stories written in a similar setting may be merged to form one whole. They are then read by the mentors at Ankur, who challenge them and help them expand their world view and sharpen their critique. Typically, the writers at Ankur work under the name of the “Ankur Writers Collective” instead of using individual author names. After the rigorous series of reviews described above and the resultant “layering” of meaning making, the singular voice of an author becomes a collective voice, which they describe as a “polyphonic critical reflection of their city.” It is this process that gives their writing an ethnographic, and perhaps even a liberatory, edge.

The writing mentors at Ankur describe their texts as “working class literature,” a genre distinct from other writings, scholarly or fictional, about life in a metropolis. They describe their process of writing as a “durational practice.” The word they use for this is *riyaaz.* A literal translation of this word in English would mean practice. However, *riyaaz* is a process that holds not only repeated practice but a sense of an endurance through time. It requires that a writer engages with her writing with a certain rigour over the years, in order to find their authorial voice and perspective. Such luxury of time and resources is not easily afforded to young working-class people. Children as young as five and six start learning to write at the centres Ankur has set up in various slum clusters across Delhi. Besides learning how to write, the children are encouraged to observe, reflect, and develop confidence to voice their observations. As they grow up, they develop into writers with a distinct literary style and use of language. Prabhat, one of the mentors at Ankur calls this ethnographic edge a function of “*doori*” or distance. Ankur’s writings emerge from the lived experiences of the people from working-class communities of Delhi. It is a community reflecting and critiquing their own lifeworld. What Prabhat describes as a polyphonic voice adds another layer of *doori* because it is no longer the subjective understanding of an individual; rather, it becomes the documentation of the perspective of an entire section of Delhi society that has built India’s capital city with its own labour.

As the above narrative shows, the stories written at the Ankur writers collective are deeply rooted in the lived experience of the people from working-class communities of Delhi. The present article draws on the perspective provided by the people who inhabit these stories, through their own worldview. Whilst the writers at Ankur do not explicitly write to engage with an academic audience, let alone use the concept of liberation medicine, I propose that their stories speak to the idea of liberation medicine in two important ways: First, at the level of content, the stories capture the many small moments of humanity that exist in the interstices of an often inhumane and exclusionary medical system. Second, at the level of process, the conditions through which the story was produced can be considered liberatory in themselves. This article then takes seriously the idea that liberation medicine, in order to be credible as a bottom-up approach (see Introduction), must be anchored in the experiences and views of those who are often excluded from the medical system and whose needs (for information, care, expertise) are often unmet (Farmer, 2003; Freire, 2000[1968]; see introduction to this issue). Listening to the stories of people like Farid Bhai and Shakeela teaches us, quite viscerally, what it feels like to encounter a hospital as a separate world, a space with its own rules and language, with maze-like pathways, and with powerful authority figures who perform their work, like magicians, behind closed doors, and who cannot be questioned. Whilst anthropologist-ethnographers have also provided detailed accounts of hospital life, including the problems of hospital care and the attempts to reform it (e.g., Rao, 2022; Solomon, 2022), hearing first-hand from “service users” gives us a phenomenologically rich account of what it is like to be a patient, or patient-relative, in these settings, and what “liberation” might mean here.

Notably, a large percentage of the Ankur writers are women of varying ages. When I asked the mentors if this was a conscious choice, they said that initially it was not. Their focus was on children. But as they grew, the boys would often drift away whilst the girls remained. The overwhelming presence of the girls has definitely brought their specific gaze to the stories. The fine mesh of relationships between women, and the ways that women come together to support each other in times of crisis, often feature in Ankur’s stories. Women regularly appear as central characters, and even when they do not, they feature as fully fleshed out individuals—their thoughts, desires, perspectives, and vision drive these stories. This is also noticeable in Farid Bhai and Shakeela’s story, which despite its central focus on Farid, reveals a careful attention to detail and emotion that characterizes the writing of Ankur’s female authors.

In 2022, I, Kavita,^10^ entered the SKP hospital gates following Lakhmi Chand, one of the mentors at Ankur. Looking around, I could see the faces of Farid Bhai and Shakeela reflected in many people who walked past me. I could feel their apprehension at being confronted by the chaos all around. SKP is a typical public hospital in India’s capital. It is funded by the Delhi state government and treats more than 7000 patients daily.^11^ Crowds of people were everywhere, and I could understand Farid Bhai and Shakeela’s confusion at not being able to work out where to begin.

One way that people deal with feeling overwhelmed when they visit a public hospital is to bring along friends and family. Thankfully, I was accompanied by Lakhmi, who was familiar with the hospital layout because he had been one of the people who had researched and written Farid Bhai and Shakeela’s story. We were both at SKP to record soundscapes in a typical big hospital in India. Before arriving, we discussed how best to record the soundscapes that would accompany this story for our multimodal website. Once we were at the hospital, Lakhmi expertly manoeuvered me through the crowds towards the Maternity Block. I asked him how the SKP management expected that people would find their way around this massive complex, and he pointed out an elaborate map printed on a board just inside the entrance to the hospital. All departments and wards were marked out with symbols, and on the right side their names were written in both Hindi and English. He also said that a constant stream of announcements came over the loudspeakers, but he was quick to add that hardly anyone referred to either the maps or the announcements. People simply asked someone for directions. So, although the hospital management does provide information on how to navigate this maze-like space, people tend to seek out human interaction rather than trying to figure out signage.

## Into the Waiting Room

*Once inside the Maternity Block, Farid and Shakeela had to wait in a long queue for registration. Seeing other pregnant women around her allayed some of Shakeela’s nervousness. Once they reached the counter, Farid handed over the doctor’s referral and Shakeela’s Aadhaar card* 12 *to the woman and filled in some forms. Then they were told to head towards the delivery ward. Shakeela took one last look at Farid and walked in. Farid was not allowed inside. He found a place to sit outside the ward and waited. Before long the guard outside the ward told him to go and sit in the waiting room and not crowd up the entrance. Farid didn’t have the heart to leave but he had no choice. He kept wondering how Shakeela was. Was she asleep, was she in pain? He called up home as he walked towards the waiting room. His mother and brother had arrived to be with his daughters whilst he was at the hospital. That was a relief. He needn’t worry about his daughters anymore. They would be looked after. Farid walked inside the waiting room.*

*The waiting room was crowded with people. There were rows upon rows of metallic benches; the back walls were hardly visible as they had been strung up with clotheslines, piled high with clothes; people were sitting, or lying, or standing everywhere. A few seats were available, but there were bags kept on them as if someone had parked them there to reserve the space for themselves. Some people seemed to be in groups whilst others were on their own. Each had a loved-one somewhere in this building complex, in a state of pain or suffering. A lady sat in a corner speaking intensely over her phone whilst tears silently rolled down her cheeks. Just next to her, another woman sat with two little children who were giggling uncontrollably. There was a family of five sitting and eating out of a steel tiffin box, and in one corner sat an old man with prayer beads. Each was hoping for a cure. As Farid stood in one corner, he took of his white skull cap, folded it and tucked it into his shirt pocket. He saw a man sitting on a red blanket sipping tea. He went up to the man and asked where he could also get a cup of tea. The man pulled out a plastic cup from his bag and poured some out for him. “My name is Mohan Das,” he said handing over the cup of tepid tea to Farid. Mohan was there for treatment of his fifteen-year-old son, who was suffering from dengue. They exchanged their stories, and Mohan showed Farid the canteen from where he could buy tea and food. But the crowds were everywhere. The canteen was also full of people eating and drinking. It could almost be mistaken for a festive occasion, with so many people having a meal, hot steam wafting from the kitchen counter, the clang and clatter of utensils. But the air smelt strange and there was a pallor which had crept into the sunlight. Looking around, Farid shook his head to clear it and remind himself that he was here for a happy occasion after all. Shakeela should be out of the hospital in a couple of days with their baby. He forced out a sort of dread which had been inching up his spine and started looking at reels on social media to cheer himself.*

*Farid went up to the guard at the maternity ward several times that day only to hear that Shakeela was well and resting. He would be summoned on the loud speaker in the waiting room if she needed anything or if the doctors needed to speak with him. In the evening his entire family came to visit him, and they sat under a tree outside the waiting room and had dinner. They had brought a pillow and a blanket for Farid. He spread this out on the floor of the waiting room and tried to get some sleep. His eyes were glued to the round clock which hung in the centre of the front wall of the room. Its luminescent hands seemed to move so slowly, in contrast to his mind, which was racing, and his heart, which was pounding. Every time his eyes became heavy, the loud speaker in the room would crackle with an announcement, and Farid would snap open his eyes lest they had some announcement for him. But there was still no news of Shakeela. Finally, in the early hours before dawn, Shakeela’s family members were called for. He ran up to the ward and heard that she had been rushed to the operating theatre. The baby was taking too long and was moving from side to side instead of descending down. A normal delivery was no longer possible, and the doctor would have to perform a C-section. He was given a bunch of forms to sign for consent and asked to wait again. He went back to the waiting room. Mohan Das, his friend from last evening, was awake and reading a newspaper. He looked up and Farid told him what had happened. “Go home and arrange for some money my friend,” he advised. “I will look after your belongings whilst you are away.”*

In anthropological literature, waiting has been explored as an important social practice, as an interplay between hoping and doubting, and as a reflection of power and powerlessness (e.g., Bourdieu, 2000; Janeja & Bandak, 2018; Hage, 2009; Nair Ambujam, 2023). Farid Bhai and Shakeela’s story vividly captures not just the uncertainty and—one could argue—violence experienced by those who have to wait, but also the sociality that emerges in times and spaces of waiting.

Inside the waiting room of the hospital, the air inside the hospital is heavy with emotion. Through Farid Bhai’s experience we see how he was immediately parted from Shakeela and all connection with her was cut. Inside the waiting room, everyone around him was in a similar state of apprehension and vulnerability. Farid Bhai never got to meet a doctor or a nurse who could tell him how Shakeela was faring. Feeling disconnected and uncertain because of a lack of information is not necessary because people are legally allowed to approach the institution and its management. But for people like Farid Bhai, who are from working-class backgrounds and have limited education, there is fear of approaching authority figures like doctors, who are hard pressed for time and under tremendous pressure to serve the burgeoning number of patients they have to treat. This fear only increases the sense of uncertainty, where even the guard of the ward can bully you into submission.

Yet, despite the sense of stagnation experienced by the people within, the waiting room also has a productive quality (Andersson, 2014; Engblom, 2023; Jeffrey, 2010) Instead of falling destitute, we see Farid Bhai connecting with other people like him, who are waiting for family members lying ill somewhere in the hospital buildings. Mohan Das becomes his friend; he shows him around the place and teaches him the ropes. All around him are other people who have been camping in the waiting room for days, as is evident from the overflowing clotheslines, along with blankets and bedding piled high all around. It is these people who lend each other a helping hand and provide both practical and emotional support.

The crackling speaker, along with the ticking hands of the clock in the waiting room, symbolize the state of being for people like Farid Bhai, who are stuck in a state of perpetual uncertainty, cut off from information and any real connection with the treatment process. Negotiating the physical space of the hospital and its various departments is difficult, as is accessing proper information about what is happening to their family member inside the ward. Unable to reach out and access information that should be rightfully theirs, they can only count the passing seconds and *wait* for information to come to them.

Liberation, when viewed in light of Farid and Shakeela’s story, is a big word; a different, more humane and approachable medical system appears to be a very distant utopia.^13^ Whilst the hospital does its best to provide physical care, emotional care grows in spaces left empty by a depersonalized medicinal system. Liberation in this story is not about revolution or protest or politicization; instead, it appears in the form of minor gestures (Manning, 2016)—offering a cup of tea, a pillow, or good advice—acts that may pass almost unperceived but which nevertheless transform the field of relations. Mohan Das recognized Farid Bhai’s pain and uncertainty when the medical staff could not find the time to take cognizance of it; he provided vital information when the doctors were out of reach. Despite desperation, tiredness, and being cramped in overcrowded hospital wards, waiting relatives establish their own “undercommons” (Harney & Moten, 2013; van der Waal, 2024), understood here as loose networks of mutual aid and sociality that emerge outside of and despite dominant institutional structures, which make hospital life more bearable and which give them strength and much-needed information, when the medical system leaves them feeling small and helpless. Illness or the shared concern for loved ones become starting points for new solidarities.

## Veterans of the Waiting Room

*Farid rushed out of the hospital. It was too early for rickshaws so he ran the entire way back home. His mother opened the door and Farid rushed into his bedroom all the whilst sharing his news. His daughters woke up and his brother’s wife got him a cup of tea and last night’s roti. They opened the metal trunk in which all important things were stored and took out whatever savings they had from a small tin. As he ate, his daughters slid into his lap. His mother stood behind him and stroked his head. His brother had quickly changed and the two went back to the hospital. As they waited outside the ward, even the guard took pity on Farid and did not shoo them back to the waiting room. Soon a doctor emerged from within. “I am so sorry, we could not save your baby. It was a still birth and your wife is critical. Her kidneys are in trouble and she will need dialysis. Arrange A+ blood as soon as you can.” It seemed like a mountain just broke over Farid’s head. He could barely comprehend what was being said to him. The doctor turned around and left hurriedly. There was no time to cry for his lost baby. He had to save his wife now. How were they to organize the blood and from where? The guard at the ward had heard the whole conversation. He came up to them and lay his arm around Farid’s shoulder. He told them to go to the main registration area and ask at the counter there. “Come back here and tell me if you don’t find the information. I will figure out a way to help you.” As the two brothers rushed towards the registration room, Mohan joined them too. On hearing everything, he said that he knew where to go. “There is a blood bank next door, let’s go there.” At the blood bank, they were directed to another blood bank at another hospital. Finally, there they were told they would get the blood, but before that they needed to donate an equal amount of blood to the bank or pay for what they needed. Farid, his brother, and Mohan Das, all three donated blood. So, thankfully they did not have to pay for the A+ blood they received.*

*Days passed slowly as Shakeela went through dialysis after dialysis. Farid’s extended family, friends, and neighbours had all stepped in to donate blood. His colleague, Ratan, had a cousin who worked in Jeevan Jyoti hospital in West Delhi. Through her help they managed to find blood at the blood bank there, without having to run around from one bank to the other. His mother and his brother’s wife would take it in turns to take care of his daughter. They would often come to the hospital during visiting hours to stroke Shakeela’s back, comb her long black hair, and wipe her tears. Her daughters would make two braids for her, like her mother did when she was little, and stealthily slip in an orange candy into her mouth whilst no one was looking. This way she could lie comfortably on her back without her hair getting into the way. Farid’s mother could be often seen in the waiting room, kneeling in prayer with her head scarf drawn over her head. They grieved for the dead baby, but they prayed for Shakeela’s return. Shakeela was slowly becoming frail. Her only desire now was to go back home. She wanted to go and lie on her own bed, put her head on her own pillow, and cook for her family and care for them like she did earlier. But this dialysis seemed to have her in its stronghold and was refusing to let go. One day Farid asked the nurse at the ward about it. “What is this dialysis?” She explained that when the kidneys fail, then blood in the body becomes contaminated, as the kidneys keep the blood clean. By doing dialysis the poisoned blood is being cleaned up and fresh blood is being added. “But how did Shakeela’s kidneys fail? How did her blood become poison?” These questions haunted Farid, and no one, not even the doctors, could give him a convincing answer.*

*Farid had become a seasoned veteran of the Waiting Room. He had a permanent corner away from the harsh blue tube lights where he would curl up and sleep in the night. He would help new patients and visitors much like he had found help in Mohan Das. He knew every corridor and floor of the hospital complex and would show people where to go when they were lost. Even the guard asked him to stand for him sometimes when he needed a quick break. Nurses would send him out on small errands. He offered people advice, tea, and hope. Much like Shakeela, he had also been admitted to the hospital.*

On further reading of Farid Bhai’s plight, we see the noose of “no-information” tightening around his neck. The questions which haunt him are bound to haunt the readers. What happened inside that delivery room? How did Shakeela lose her baby? What really happened in there that led to the loss of a life, a life that never saw the light of day?

Farid Bhai’s human connections in the world are pitted against a rigid and overburdened medical system. The only information he receives from a qualified doctor is to first hear about Shakeela’s surgery, then the passing away of his baby and the demand to organize A+ blood for the mother. There is no one in place to offer him any further information or explanation. He has many questions, but the medical system remains elusive, operating behind closed doors. In contrast, his human connections are there to stroke his back and bring him strength. Even the hard-hearted guard puts his arms around his shoulders and offers him help on hearing the news of the baby passing away. His family, friends, and colleagues rally around him to give him support. The social network in which he lives tries to hold him together. Yet, the “undercommons” in this case can only go so far: they can provide knowledge, care, and empathy, but they cannot save the baby.

The image of two separate worlds sharpens as we continue to read Farid and Shakeela’s story. One, the “known” world, is made up of people who inhabit a similar class position and who can help each other based on their knowledge of what it feels like to be ill and need medical care *as someone of this* “*world*.*”* The other, the distant world of the hospital, evokes fear as much as hope, and most people enter it only as a last resort, when all forms of proximate care (including that by community-based physicians, like Shakeela’s doctor at the local dispensary) and all strategies for avoiding illness have failed. For Farid Bhai, Shakeela, and for the Ankur writers who captured their story, neither the hospital nor the people working there are villains; the hospital world simply feels radically inaccessible and opaque. Often the causes of sickness and the pathways to cure are hard to disentangle. Seen through Farid Bhai’s perspective, Shakeela had been captured in the stronghold of “dialysis.” In his eyes, dialysis is part of the sickness that is keeping Shakeela in the hospital and making her unwell. When he tries to understand this phenomenon by reaching out to one of the nurses, she explains that dialysis is a process which is doing the work of Shakeela’s kidneys, which have failed. But she does not explain why they failed. This only adds to Farid Bhai’s confusion, leaving him wondering whether dialysis is really part of the cure. All he sees is that dialysis, like a disease, is responsible for draining Shakeela day by day and is forcing her and her family to endure emotional and financial suffering (cf. Hamdy, 2008).^14^

In this context, I would like to introduce an important point of departure, one that I have often heard from Lakhmi and the other writers at Ankur, which is particularly relevant to Farid Bhai and his experiences. For the working-class people in Delhi, sickness is defined only by the inability to get up and go to work. Lakhmi once told me: “The day we feel we are unable to get to work and earn our daily wages, we go to the nearest and most efficient doctor we can find, and that is when we may call ourselves sick. Not before that.” So, for the labourers in Delhi, the condition of being sick is not, or not primarily, defined by the symptoms but rather by their inability to bring in their daily wages. For most working-class people like Farid Bhai, missing a day’s work may mean they cannot light the kitchen fire that evening. So, when we read that, much like Shakeela, Farid had also been admitted to the hospital, then this sentence gives us a deep insight into the everyday lives of the people we are reading about. When we try and unpack this sentence, we see that it becomes irrelevant what was making Shakeela ill. The fact was that she was becoming frail with every passing day as she went through one dialysis after the other. Along with Shakeela, Farid Bhai, who was also in a perpetual state of waiting, was not able to go back to his life and his work—thereby he had also been “admitted to the hospital.” Because he was unable to earn his livelihood, he had also become sick. And by extension, their daughters, who were dependent on them, had perhaps also been drawn into this circle of sickness.

In the story thus far, one finds resonance with anthropologist Nancy Scheper-Hughes (1991) and others (e.g., Scheper-Hughes & Lock, 1987) when they argue that bodily disease can often not be separated from the social suffering it reflects and induces; the individual body is deeply entangled with a social body, and the sickness of one of these bodies has immediate consequences for the other. When Shakeela, and by extension Farid Bhai and their family suffer, an entire community of people are drawn into their ring of experiences. As their friends, acquaintances, and neighbours share their pain, so do they share their experience of negotiating the medical system. This further extends the fear instilled in the heart of people in the community for a medical world that is vast and seemingly beyond their ability to understand and access.

## Leaving

*After a year’s struggle, Farid had sold his small house and his blood, his daughters were being supported by his brother, he had to sell their land in the village, his mother’s forehead had developed a permanent scar from the countless times she had bowed her head in namaz.* 15 *Shakeela had developed fluid in her spine; her legs had become lifeless. How much more could she endure? One day Shakeela left the hospital and this world forever more. Farid’s heart and mind had been battered. He was almost relieved that Shakeela had passed away and beyond all pain. He also left the hospital and went back home to his mother and daughters.*

*Even now, people who know Farid come to him for help if they need support to negotiate their way across SKP Hospital. He tells them which kind fellow to connect with, in which department, who might help them ease their way through the confusing hospital corridors. In some rare cases, Farid will get up from his sewing machine, don his white skull cap, and accompany people to the hospital so that they may find cure, something which alluded Shakeela and himself.*

This story tells us about the loss of two lives and has particularly resonated with me since the first day that I read it. For much like Shakeela, I am also a mother who had been in a similar situation. When my baby did not descend, I also had surgery in an Indian hospital. But the outcome of my experience was very different from that of Shakeela’s. In Shakeela and Farid Bhai’s experience, I see at play the class and social structure of Indian society. But one thing that endures through this extremely tragic, almost cruel tale is a story of hope. Farid Bhai lost his house, his land, his baby, and his wife. Yet he did not lose his humanity. Just as he had found help first through the compounder, then through Mohan Das, and through countless other friends and family members, he continues this web of hope by extending his hand to others. He now seems to act as an invisible key to the gates of the SKP hospital, helping ordinary people access and unlock a world that—without help—often engulfs them. Farid Bhai might not have managed to save his wife, but he has surely taken on the mantle of supporting countless others, even though he is not a medical practitioner himself. At the beginning of the story, he had set out from his home with his wife to bring back their baby, with his white skull cap firmly perched on his head. But somewhere along the line, whilst he waited for information about Shakeela, he had folded it and tucked it away in his pocket. Now, though on rare occasions, we are told, he dons his white cap and sets out to show others the way through the labyrinth of the hospital. Through this story, we are able to see that people are not devoid of agency, and there is always hope for social justice as long as there are people who are willing to take a step towards it. Humanity, in the face of adversity, uncertainty, and tragedy is provided not by the medical institutions but those who are willing to give and do care work.

Shakeela’s death led Farid Bhai to learn what he had already known before: that for people like him and Shakeela, hospitals would always remain separate, and largely inaccessible, worlds. Yet the encounters with the medical system and the differently positioned people within it still led to changes, however small. Their story shows that even in contexts in which cure is out of reach, care and solidarity are meaningful. Sometimes, radical care rests, perhaps can only rest, in “minor gestures” (Manning, 2016).

In India, as Rao’s (2022) work has shown, even well-intentioned attempts to reform state medical care have not been successful for the poor. In fact, what the burgeoning field of hospital ethnography—in India and beyond (e.g., Abadía-Barrero, 2022; Livingston, 2012; Pinto, 2014; Varma, 2020)—suggests that hospitals are often simultaneously places of care and places of violence, places that tend to reproduce the structural inequalities and forms of discrimination found in society at large. In the face of this literature, it would seem naïve to assume that there was an easy road to liberation, especially given the global trend of neoliberal policies and related budget-cuts in the healthcare sector.

However, what emerges from the story of Farid Bhai and Shakeela is that people who are powerless do not just succumb to situations that are beyond their control. They might not be able to smash a structure of embedded power, but they do try to find lateral means of negotiating through it. This is visible through the networks of care and support that emerge in the form of family, friends, and strangers who help Farid Bhai and his family. Farid Bhai himself also becomes a person who doesn’t succumb to his circumstances but instead helps others fight and negotiate the system. Farid Bhai’s name has been anonymized, yet his experiences are real.

Not all experiences end the way Farid Bhai’s did. Yet, there are reasons why the collective chose to document this story and present it to the world. In their own words, they describe this as “*jijeevisha.*” The closest translation I can find for this is “the lust for life.” The act of writing for the collective is itself an act of liberation; the writers do not look to a higher order to liberate them. When seen in the context of liberation medicine therefore, whilst the Ankur writers do desire a system that is just and fair, they want to point out that people like Farid Bhai, people like themselves, find ways to negotiate a path through the system. For them, every day is a fight for survival. Sometimes they fall, but every day they get up and challenge it.

The story of Farid Bhai and Shakila, along with the many other stories that the Ankur Writers Collective have penned from their own life experiences, portray people confronted by vast, daunting systems. Yet they don’t always crumble with fear and remain victims of this system. Instead, they find ways to traverse it even in the face of grief and loss. By helping each other, people manage to create a world worth living in and produce forms of care in the absence of utopian care institutions. These networks of care are easy to overlook in the search for perfect institutions, but there are no perfect institutions without people caring, communicating, and making themselves available to others. Liberation can perhaps grow only where networks of care come together.

## Footnotes

1 All names of characters in this ethnographic story have been anonymized.

2 The name of the hospital has been anonymized.

3 A (usually) metal box used in the Indian subcontinent to carry small meals, especially lunch.

4 An Indian whole wheat flat bread cooked on a griddle.

5 A small vehicle with three-wheels and an engine, typically with open sides, which is often used in the Indian subcontinent as a makeshift taxi (also referred to as an auto).

6 A doctor’s assistant who dispenses medicines but does not have the qualifications to prescribe them. In India, they can be found providing assistance in big hospitals or busy doctor’s clinics.

7 A term of respect used in Northern parts of India, to address people of a higher social stature.

8 Literally meaning “brother”; a polite form of address used for a man in northern parts of India.

9 https://ankureducation.net/childrens-programme/learning-collective-7/.

10 I, Kavita Dasgupta, am the curator and editor of ‘Ticketless Travellers’. https://ticketless-travellers.info, a multimodal website curating the writings of The Ankur Writers Collective. I have worked closely with the writers from the Collective to review the stories, visualize them, trained the writers to create media content and translated all their original stories and texts written in Hindi to English. I also happen to have been born and raised in Delhi for the first forty years of my life.

11 This news report gives a sense of the massive scale of public hospitals such as SKP: https://timesofindia.indiatimes.com/city/delhi/lok-nayak-eyes-new-high-with-a-22-storey-centre/articleshow/77859860.cms, accessed 15 October 2024. For a detailed ethnographic account of the ‘traffic’ in such hospitals, see Solomon (2022).

12 Aadhaar is a twelve-digit unique identity number that can be obtained voluntarily by all residents of India, based on their biometrics and demographic data. Aadhaar is used for various governance purposes, including health insurance (Rao & Vijayanka, 2019).

13 Rao’s (2022) research describes the “curiously utopian” attempts by Indian policy makers to improve hospital care and health coverage despite the chronically underfunded medical system, persistent high social inequality, and recurrent failures of medical reform. Whilst the problems of the current system are well known and different, and sometimes quite far-reaching reforms have been implemented, the latter have not resulted—nor are likely to result—in significant improvements for poor people.

14 In her research with poor Egyptian kidney disease patients, Sherine Hamdy (2008) describes the deep mistrust these patients feel towards state-provided medical care. She shows how, for these patients and their families, both the failing kidneys and the dialysis machines that are supposed to help them are implicated in a larger “political aetiology” of disease.

15 Namaz is the word used on the Indian subcontinent for the prayers offered five times a day by Muslims.
